# New insights on Noonan syndrome’s clinical phenotype: a single center retrospective study

**DOI:** 10.1186/s12887-022-03804-2

**Published:** 2022-12-24

**Authors:** Francesco Baldo, Alice Fachin, Beatrice Da Re, Elisa Rubinato, Marco Bobbo, Egidio Barbi

**Affiliations:** 1 Department of Medicine, Surgery and Health Sciences, University of Trieste, Trieste, Italy; 2 Institute for Maternal and Child Health IRCCS Burlo Garofolo, Trieste, Italy

**Keywords:** Noonan syndrome, RASopathies, Phenotype, Diagnosis, Genetic diagnosis

## Abstract

**Background:**

Noonan syndrome (NS) is a clinically and genetically heterogeneous disorder. Since its clinical phenotype is often mild and difficult to differentiate from other syndromes, its diagnosis can be challenging and its prevalence in the pediatric population is most certainly underestimated. The difficulty in identifying Noonan syndrome is also increased by the fact that genetic tests are currently not able to detect an underlying mutation in around 10% of the cases.

**Methods:**

This is a retrospective, observational study conducted at the Institute for Maternal and Child “Burlo Garofolo” in Trieste, Italy. We recruited all the patients with clinical and/or genetic diagnosis of NS who were evaluated at the Department of Pediatrics between October 2015 and October 2020. Statistical analyses were performed with IBM SPSS Statistics software. The association between discrete variables has been evaluated through chi-squared test, indicating statistically significant p with Pearson test or Fischer test for variables less than 5.

**Results:**

We recruited a total of 35 patients affected by Noonan syndrome. In 24 patients (75%) we identified an underlying genetic substrate: 17 patients had a mutation on PTPN11 (61%), 2 in SOS1, KRAS and SHOC2 (7% each) and only 1 in RAF1 (4%). 25% of the subjects did not receive a genetic confirm. As for the phenotype of the syndrome, our study identified the presence of some clinical features which were previously unrelated or poorly related to NS. For example, renal and central nervous system abnormalities were found at a higher rate compared to the current literature. On the contrary, some features that are considered very suggestive of NS (such as lymphatic abnormalities and the classical facial features) were not frequently found in our population.

**Conclusions:**

In our analysis, we focused on the main phenotypic features of NS, identifying various clinical manifestation that were not associated with this genetic condition before. This could be helpful in raising the knowledge of NS’s clinical spectrum, facilitating its diagnosis.

## Background

Noonan syndrome (NS) is one of the most common genetic disorders, with an estimated prevalence of 1 in 1.000 to 2.500 newborns [1]. NS is part of the so-called “RASopathies”, a group of genetic syndromes caused by germline mutations in genes that encode components of the Ras/mitogen-activated protein kinase (MAPK) pathway, which plays a critical role in cell differentiation, proliferation, and survival [2]. NS is mostly inherited in an autosomal dominant manner, with variable expression and penetrance [3]. In more than 50% of cases, missense mutations in PTPN11 gene on chromosome 12 are responsible for this condition. Other well-known causative genes are SOS1, RAF1, RIT1, KRAS, BRAF, NRAS and LZTR1 [4]. LZTR1, for example, has been identified in up to 10% of NS individuals, showing either autosomal dominant or autosomal recessive inheritance [5]. As a matter of fact, the knowledge of NS basis is steadily expanding, and more and more genes, such as RRAS2, MRAS, SPRED2 and SOS2, have now been included in its genetic spectrum. Nevertheless, approximately 10% of patients tested for NS lacks a genetic diagnosis, suggesting that other genes might be involved [6]. In any case, the diagnosis remains clinical and based on the Van der Burgt criteria [7].

The most common clinical features in NS include:

*-* Craniofacial dysmorphisms, such as triangular shaped face, tall forehead, hypertelorism, ptosis, low-set, and posteriorly angulated ears. NS with PTPN11 mutation is most strongly related with these features [8].

- Growth defects. Birth weight and length might be within the normal range for newborns. The typical growth retardation emerges after the first year of life and it is more common and severe in subjects with PTPN11 mutations [9, 10]. PTPN11 mutations are also associated with a lower response to GH replacement therapy [11].

- Cardiac involvement, which affects about 80%, usually represented by pulmonary valve stenosis (PVS, 60–70%), hypertrophic cardiomyopathy (HCM, 20–30%) and atrial septal defects (ASD, 10–30%). The incidence of PVS is higher when PTPN11 is mutated, while HCM is most frequent in patients who present RAF1 and RIT1 mutations [12].

- Neurodevelopmental abnormalities, including developmental delay, executive function deficits, mild intellectual disability (which could be more severe in KRAS mutations), autism spectrum disorder, and attention deficit hyperactivity disorder. Structural brain abnormalities have also been described [13].

- Genitourinary tract malformations, such as kidney pyelectasis, ectopia, or duplicating collecting systems, occur in approximately 10% of patients [14]. Cryptorchidism has been described in 60–80% of males with NS [15].

- Lymphatic abnormalities, that can occur in more than 20% of NS subjects. Lymphedema is the most common finding; less frequent, but more severe findings are chylothorax and ascites. Pre-natal features suggestive for lymphatic anomalies include increased nuchal translucency, cystic hygroma and polyhydramnios [16, 17].

- Hematologic manifestations and malignancies. Abnormal bleeding and bruising have been described in NS (especially prolonged aPTT, due to mild to severe deficits of coagulation factors of the intrinsic pathway) [18, 19]. An increased risk of malignancies (RR 8.1, 95% CI 3.5–16) has been described in children with NS, especially concerning the incidence of juvenile myelomonocytic leukemia (JMML), myelodysplastic syndrome (MDS), acute lymphoblastic leukemia (ALL) and neuroblastoma (NBS) [20, 21].

- Ocular abnormalities including strabismus, refractive errors, amblyopia, and nystagmus have been described in up to 95% of NS [22].

- Hearing impairment, that occurs in up to 40% of the subjects. It may be conductive (the most common, mainly due to recurrent media otitis), neurosensorial, or mixed and of any severity [23].

- Cutaneous manifestations: ectodermal abnormalities such as severe follicular keratosis over extensor surfaces and face are typical of NS, especially in SOS1 mutations; multiple lentigines and other skin pigmentation disorders may occur [24].

Another element that adds complexity to the wide and heterogenous spectrum of symptoms in NS is the fact that some RASopathies are clinically closely correlated to it, despite being caused by different pathological mechanisms. This is the case of Noonan syndrome with multiple lentigines (NS-ML), also known as LEOPARD syndrome, and Noonan syndrome-like disorder with loose anagen hair (NS-LAH), also known as Mazzanti syndrome. Like NS, NS-ML is also caused by PTPN11 variants, but the molecular pathway affected is different, since the upregulation involves PI3K-AKT-mTOR signaling and not MAPK [25]. On the other hand, NSL-AH is caused by mutations in a different gene, SHOC2 [26].

In consideration of the wide clinical heterogeneity of NS and of the continuous reports of new features that are expanding its phenotype, we reviewed our center’s cohort highlighting the most unusual presentations, to widen the available knowledge on NS.

A genotype-phenotype correlation has been identified for many of these clinical features of NS, as reported in Table 1*.*

**Table 1:** Noonan syndrome causative genes and associated phenotype

***Gene***	***Phenotype***	***Genotype-phenotype correlation described in literature***
PTPN11	NS	Classical NS, due to PTPN11 gain-of-function variants. In this the highest incidence of pulmonary valve stenosis is described. In some cases, JMML risk is increased
	NS-ML	Mainly caused by PTPN11 loss-of-function variants, frequently associated with multiple lentigines, facial dysmorphism and ECG conduction abnormalities
SOS1	NS	Less short stature and intellectual disability. Follicular keratosis is common
SOS2	NS	Similar to SOS1 phenotype, with higher incidence of lymphatic abnormalities
RAF1	NS, but variable	Highest incidence of hypertrophic cardiomyopathy
RIT1	NS, but variable	Normal growth, and intellect. High incidence of cardiac abnormalities; particularly, hypertrophic cardiomyopathy is common
LZTR1	NS, but variable	In autosomal recessive forms, lymphatic abnormalities and hypertrophic cardiomyopathy are common. In autosomal dominant forms, phenotype is generally milder
NRAS	NS	Classical NS
RRAS2	NS	Classical NS
MRAS	NS	Classical NS with common hypertrophic cardiomyopathy
SHOC2	NS-LAH	Sparse hair, hyperpigmented skin, eczema, keratosis pilaris; short stature is a common feature
KRAS	Some NS/CFC	Higher rate of intellectual disability
BRAF	Some NS/CFC	Intellectual disability is common, as for skin and hair abnormalities

*CFC: Cardiofaciocutaneous syndrome

## Methods

This is a retrospective observational study conducted at the Institute for Maternal and Child “Burlo Garofolo” in Trieste, Italy. We recruited all the patients with clinical and/or genetic diagnosis of Noonan syndrome who were evaluated at the Department of Pediatrics between October 2015 and October 2020. Subjects with variants of Noonan syndrome, such as Noonan syndrome with loose anagen hair (NS-LAH) and Noonan syndrome with multiple lentigines (NS-ML), were also included.

For each patient we collected the following data: gender, age, type of disease (classic Noonan, NS-ML or NS-LAH), type of diagnosis (genetically confirmed or not), type of mutation, and phenotypic features. Among the latter, we specifically investigated the presence of heart disease, with electrocardiogram (EKG) and echocardiographic features.

Genetic analyses were performed either with Whole Exome Sequencing (WES), multigene panels with Next Generation Sequencing (NGS) techniques, or by single gene sequencing of PTPN11, depending on the case and the availability of the analysis method at the time of the diagnosis. In our Institute, NGS panels include the following genes: PTPN11, SOS1, RAF1, SHOC2, BRAF, MAP 2 K1, MAP 2 K2, RIT1, NRAS, KRAS, HRAS, RRAS, SOS2, LZTR1. Currently WES is our standard genetic analysis in the suspect of RASopathies. If genetic tests resulted negative, the clinical diagnosis was made according to the Van der Burgt criteria.

To understand if a specific clinical feature was already associated with Noonan syndrome, or at least previously reported, we performed research on Pubmed by typing “Noonan” or “Noonan syndrome” and that feature (for example, Noonan AND hypertelorism). If no manuscripts appear after the research, we considered that feature as previously unrelated.

Statistical analyses were performed with IBM SPSS Statistics software. Discrete variables are presented as absolute frequencies and percentages of the total, while continuous variables are presented as mean value ± standard deviation. The association between discrete variables has been evaluated through chi-squared test, indicating statistically significant p with Pearson test or Fischer test for variables less than 5. A statistical association with *p* < 0.05 was considered significant.

## Results

We recruited a total of 35 patients affected by Noonan syndrome, Noonan syndrome with loose anagen hair (NS-LAH) and Noonan syndrome with multiple lentigines (NS-ML). Three of them were excluded from the study due to the absence of complete medical information. Of the remaining 32 individuals, 18 were males (56%) and 14 were females (44%), with a mean age at the time of data analysis of 14 years of age (range 1–26). 29 patients (91%) were diagnosed with classic Noonan Syndrome, 2 (6%) with NS-LAH, and 1 (3%) with NS-ML.

**Fig. 1:**
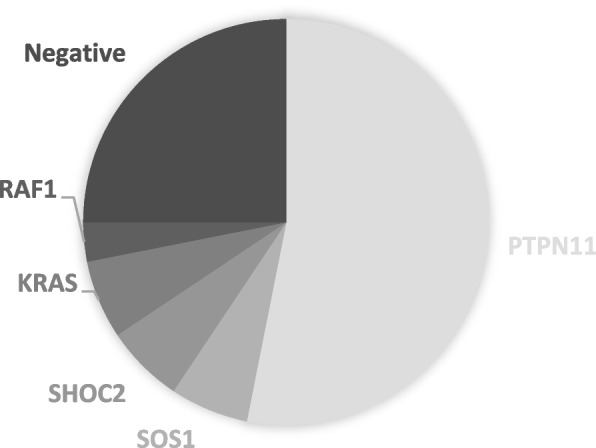
Patients’ genetics in our study cohort

In 24 patients (75%) we identified an underlying genetic substrate: 17 patients had a mutation on PTPN11 (61%), 2 in SOS1, KRAS and SHOC2 (7% each) and only 1 in RAF1 (4%) *(*Fig. 1*).* The 2 patients with SHOC2 mutations presented a NS-LAH phenotype, while the only patient with NS-ML had a mutation on PTPN11, as already known from the medical literature [27].

All the pathogenic variants available have been reported in Table 2*.* In 6 patients the variant is missing, due to incomplete data.

Among the remaining 8 children with clinical diagnosis and negative genetic tests (25%), 6 (19%) underwent only PTPN11 sequencing and 2 (7%) presented a negative panel for RASopathies.

**Table 2:** Genetic variants identified in the study population

**PTPN11**	
- NM_002834.5(PTPN11):c.417G > C (p.Glu139Asp)	
- NM_002834.5(PTPN11):c.1510A > G (p.Met504Val)	
- NM_002834.5(PTPN11):c.172A > G (p.Asn58Asp)	
- NM_002834.5(PTPN11):c.1391G > C (p.Gly464Ala)	
- NM_002834.5(PTPN11):c.214G > T (p.Ala72Ser)	
- NM_002834.5(PTPN11):c.214G > T (p.Ala72Ser)	
- NM_002834.5(PTPN11):c.922A > G (p.Asn308Asp)	
- NM_002834.5(PTPN11):c.1403C > T (p.Thr468Met)	
- NM_002834.5(PTPN11):c.1471C > T (p.Pro491Ser)	
- NM_002834.5(PTPN11):c.205G > C (p.Glu69Gln)	
- NM_002834.5(PTPN11):c.836A > G (p.Tyr279Cys)	
- NM_002834.5(PTPN11):c.417G > C (p.Glu139Asp)	
- NM_002834.5(PTPN11):c.218C > T (p.Thr73Ile)	
**SOS**	
- NM_005633.4(SOS1):c.1132A > G (p.Thr378Ala)	
**RAF1**	
- NM_002880.4(RAF1):c.770C > T (p.Ser257Leu)	
**SHOC2**	
- NM_007373.4(SHOC2):c.4A > G (p.Ser2Gly)	
**KRAS**	
- NM_004985.5(KRAS):c.40G > A (p.Val14Ile)	
- NM_004985.5(KRAS):c.101C > T (p.Pro34Leu)	

In terms of phenotypic features, all the data collected have been divided per area of interest (Tables 3 and 4*)*. As for the characteristics of the facies, the most frequent findings were low-set and posteriorly rotated ears (12 patients, 38%), hypertelorism (8 patients, 25%), eyelid down-slanting (8 patients, 25%) and eyelid ptosis (5 patients, 16%). We also identified 5 patients with prominent and broad forehead (16%), 5 patients with elongated filter (16%), and 3 with triangular facies (9%). Pterygium colli was present in 6 patients (19%). Short stature (defined as a height 2 standard deviations or more below the mean for children of that sex and chronological age) was found in 24 children (75%).

As regards the tumoral risk, 2 patients (6%) were diagnosed with malignancies: particularly, we identified one case of dysembryoplastic neuroepithelial tumor (DNET), and one case of juvenile myelomonocytic leukemia (JMML). In both cases, PTPN11 was mutated, consistently with the current literature. We also recognized 2 patients (6%) with benign, non-cancerous, tumors: both patients had giant-cell tumors (affecting the left tibia and the nerve sheath of the first finger of the right hand, respectively); in the respective cases, PTPN11 and SOS1 mutations were identified.

Among the skeletal alterations, scoliosis was detected in 7 patients (22%), pectus excavatum in 10 (31%), and pectus carinatum in 3 of them (9%).

Cryptorchidism was identified in 11 patients (61%) and renal abnormalities in 10 (31%). Among the latter, 7 patients had kidney pyelectasis, while renal tubular acidosis, polycystic kidney disease (PKD) and horseshoe kidney were identified in one patient each.

Lymphatic abnormalities were present in 6 children (19%): 2 patients had hydro/chylothorax, 2 had pulmonary lymphangiectasia, one had hydrops fetalis and one had a cystic hygroma.

Coagulation disorders were identified in 24 cases (75%). We found out increased INR in 4 cases (13%), while prolonged aPTT was described in 2 cases (6%). We identified a specific coagulation factor deficiency in 5 cases (16%): particularly, factor V was deficient in 1 patient (3%), factor VII in 4 (13%), factor IX in 1 (3%), factor X in 3 (9%), factor XI in 2 (6%) and factor XII in 3 (9%). Protein C and S deficiency, high D-dimer levels and thrombocytopenia were found in 1 patient each (3%).

As for the gastrointestinal manifestations, we recorded 2 cases of feeding difficulties and one of gastroesophageal reflux disease (GERD).

**Table 3:** Dysmorphic features

Dysmorphic features	N° Patients	%
Low-set and posteriorly rotated ears	12	38%
Eyelid down-slanting	8	25%
Hypertelorism	8	25%
Prominent and broad forehead	5	16%
Eyelid ptosis	5	16%
Elongated filter	5	16%
Triangular facies	3	9%

**Table 4:** Clinical features. Coagulation defects were found out in 24 cases (multiple defects could be found in a single patient)

Clinical features	N° Patients	%
Coagulation defects	24	75%
Short stature	24	75%
Cryptorchidism	11	61%
Pectus excavatum	10	31%
Renal abnormalities	10	31%
Scoliosis	7	22%
Pterygium colli	6	19%
Refractive defects	6	19%
Lymphatic abnormalities	6	19%
Hypoacusia	5	16%
Psychomotor delay	4	13%
Pectus carinatum	3	9%
Intellectual disability	3	9%
Gastrointestinal symptoms	3	9%
Malignancies	2	6%
Benign tumors	2	6%
Hypothyroidism	2	6%
Arnold Chiari malformation	2	6%
Dental agenesis	1	3%
Choledochal cysts	1	3%

With regards to the cardiological features of the syndrome *(*Table 5*)*, pulmonary stenosis was the most frequent abnormality since it was identified in 11 children (34%). Signs of hypertrophic cardiomyopathy (HCM) were found in 3 patients (9%): one of them presented an obstructive form with a RAF1 mutation, which is known for being associated with this feature. Atrial septal defect (ASD) and ventricular septal defect (VSD) were found in 5 patients each (16%), as well as mitral regurgitation. As for the EKG characteristics, right axis deviation was identified in 13 patients (41%) and left axis deviation in 2 (6%). Right branch block was present in 6 patients (19%), first-degree atrioventricular block in 5 (16%) and repolarization abnormalities in 5 (16%). The association between pulmonary stenosis at the echocardiogram and right axial deviation was statistically significant (p 0.02). Overall, we found no statistically significant associations between the cardiological features of the patients and the underlying genetic mutations.

**Table 5:** Cardiological features. ASD: atrial septal defect; VSD: ventricular septal defect; AV: atrioventricular; SAM: mitral valve systolic anterior motion; AVCD: atrioventricular canal defect; TOF: tetralogy of Fallot

Cardiological features	N° Patients	%
Right axis deviation	13	41%
Pulmonary valve stenosis	11	34%
Right branch block	6	19%
ASD	5	16%
VSD	5	16%
Mitral valve regurgitation	5	16%
First-degree atrioventricular block	5	16%
Repolarization abnormalities	5	16%
Pulmonary valve dysplasia	3	9%
Hypertrophic cardiomyopathy	3	9%
Hypertrophy EKG signs	3	9%
SAM	2	6%
AVCD	2	6%
TOF	2	6%
Left axis deviation	2	6%
AV valves dysplasia	1	3%
Mitral valve prolapse	1	3%

*Previously unreported clinical findings.*

In addition to the previously described features, which are already known as part of the Noonan spectrum, we identified various clinical manifestation that were not associated with this genetic condition before *(*Table 6*)*. The most frequent of them were winged shoulder blades, hypotonia and feet abnormalities, which were identified in 3 subjects each (9%).

Angiomas, single atrioventricular valve, clinodactyly and Arnold Chiari malformation were identified in 2 individuals (6%). Finally, high arched palate, corpus callosum agenesis, epilepsy, erythromelalgia, Raynaud’s phenomenon, choledochal cysts, laryngomalacia, microcephaly, umbilical hernia, ischemic heart disease and osteopenia have been found in one patient (3%) each.

**Table 6:** Other findings, associated mutations and percentage of expression in our cohort

Other clinical features	N° Patients	Associated mutations (number of cases)	%
Winged shoulder blades	3	PTPN11 (2) SOS1 (1)	9%
Hypotonia	3	PTPN11 (2) RAF1 (1)	9%
Feet defects	3	2 PTPN11 (2) Negative genetic test (1)	9%
Angioma	2	PTPN11 (1) SOS1 (1)	6%
Single atrioventricular valve	2	PTPN11 (1) Negative genetic test (1)	6%
Clinodactyly	2	PTPN11 (2)	6%
High arched palate	1	PTPN11 (1)	3%
Corpus callosum agenesis	1	Negative genetic test (1)	3%
Epilepsy	1	RAF1 (1)	3%
Erythromelalgia	1	SOS1 (1)	3%
Raynaud’s phenomenon	1	Negative genetic test (1)	3%
Laryngomalacia	1	KRAS (1)	3%
Microcephaly	1	PTPN11 (1)	3%
Umbilical hernia	1	Negative genetic test (1)	3%
Ischemic heart disease	1	Negative genetic test (1)	3%
Osteopenia	1	Negative genetic test (1)	3%

## Discussion

This study highlights some previously unreported or rarely mentioned findings in Noonan Syndrome.

Noonan Syndrome is one of the most common genetic conditions. However, due to a clinical phenotype that is often mild and difficult to differentiate from other syndromes, its diagnosis can be challenging and its prevalence in the pediatric population is most certainly underestimated. The difficulty in identifying Noonan syndrome is increased by the fact that genetic tests are currently not able to detect an underlying mutation in around 10% of the cases [6]. Thus, the key to an early diagnosis of Noonan syndrome relies on having a high suspicion of the syndrome in presence of specific diagnostic clues. Some of them are already well-known. Congenital heart defects, for example, are typical of this condition, as well as short stature, chest and spine abnormalities, cryptorchidism, and coagulation disorders. Our study suggests other features of this syndrome that have just occasionally reported or not even described yet. Renal abnormalities, for example, were found in 31% of our patients, a percentage much higher than the 10% prevalence reported in the literature [28]. Hypotonia would be another underestimated sign of Noonan syndrome, since it was previously described only in the neonatal and perinatal period [29].

As for Arnold Chiari malformation, this was reported in subjects with NS in just 11 cases. Interestingly, in our small-size population, this rare abnormality was identified in 2 children (6%), which is a higher rate compared to the general population (0.1%) [30–33].

Epilepsy, which we identified in one of our patients, was previously associated only with Cardiofaciocutaneous syndrome (CFC), and not with NS [34].

Another unusual clinical feature we found in one of our children is choledochal cysts, which was previously described just once in a patient affected by NS [28]. Nonetheless, this is a relevant feature, in consideration of a potential malignant degeneration.

On the contrary, some features that are very suggestive of Noonan syndrome and of RASopathy in general were not frequently found in our study. Lymphatic abnormalities, for example, are described in up to 50% of the patients with Noonan syndrome, while only 19% of our population presented them [35]. The classic facial features (triangular facies, eyelid ptosis) and the cognitive retardation were also poorly represented in our population. The lack of classic Noonan features can be interpreted as a tangible sign of the wide clinical spectrum of the syndrome that we described above. However, being this a retrospective study, it can also be caused by numerous other variables, from the young age of some patients to an insufficiently long follow-up, or even to the lack of clinical data when obtained from other Centers who later referred the patient to ours. An important role could have been played by the genetic composition of our cohort, (i.e. the percentage at which every genetic mutation is present in our group). For example, some causative genes of NS were poorly represented, and others lacked at all in our population, hence the different prevalence of some clinical features. However, this element alone cannot explain the entirety of our findings, since, for example, due to higher prevalence of PTPN11 we should have noticed a higher percentage of patients characterized by the typical facial dysmorphism of NS.

In this study, we gave a particular attention to the cardiological features of Noonan syndrome. As reported by other authors in the past, we confirm not only that congenital heart defects are a key point of this condition, but also that EKG abnormalities are very common in these subjects. However, they did not necessarily correlate with a cardiac structural aberration [36]. Thus, EKG abnormalities might represent a marker of suspicion for Noonan syndrome, but not a marker for the severity of its cardiac involvement.

Moreover, we tried to correlate the clinical phenotype of our patients with their genotype, without finding any statistically significant association.

This study certainly has some limits, the main one being the small size of our population. Another one is the fact that some rare and already discovered clinical features of NS might have been reported but did not appear in our Pubmed research, despite our efforts and meticulous analysis.

Furthermore, being a retrospective study, we had to exclude some subjects from our cohort due to the absence or the incompleteness of medical documentation.

## Conclusions

This study confirms the high phenotypical heterogeneity of NS, highlighting the fact that some clinical findings, which have not been frequently described in this condition before, could be helpful in leading to the diagnosis of NS, especially in subjects with a mild phenotype. A broader, deeper knowledge of the clinical manifestations of this condition is even more relevant considering that up to 10% of the patients lacks a genetic diagnosis, and thus also subtle features may represent the key to a precocious identification of the syndrome.

Hopefully, the phenotypic features that we have described here will be helpful in raising the knowledge of the NS clinical spectrum and facilitate its diagnosis.

## Data Availability

All data generated or analyzed during this study are included in this published article.

## Abbreviations

ALL: Acute lymphoblastic leukemia
ASD: Atrial septal defects
CFC: Cardiofaciocutaneous syndrome
DNET: Dysembryoplastic neuroepithelial tumor
EKG: Electrocardiogram
GERD: Gastroesophageal reflux disease
HCM: Hypertrophic cardiomyopathy
JMML: Juvenile myelomonocytic leukemia
MAPK: Mitogen-activated protein kinase
MDS: Myelodysplastic syndrome
NBS: Neuroblastoma
NGS: Next generation sequencing
NS: Noonan syndrome
NS-LAH: Noonan syndrome with loose anagen hair
NS-ML: Noonan syndrome with multiple lentigines
PVS: Pulmonary valve stenosis
VSD: Ventricular septal defect
WES: Whole exome sequencing
